# Assessment of Knee Cartilage Stress Distribution and Deformation Using Motion Capture System and Wearable Sensors for Force Ratio Detection

**DOI:** 10.1155/2015/963746

**Published:** 2015-08-31

**Authors:** N. Mijailovic, R. Vulovic, I. Milankovic, R. Radakovic, N. Filipovic, A. Peulic

**Affiliations:** ^1^Faculty of Engineering, University of Kragujevac, Sestre Janjic 6, 34000 Kragujevac, Serbia; ^2^Bioengineering Research and Development Center Kragujevac, Prvoslava Stojanovica 6, 34000 Kragujevac, Serbia

## Abstract

Knowledge about the knee cartilage deformation ratio as well as the knee cartilage stress distribution is of particular importance in clinical studies due to the fact that these represent some of the basic indicators of cartilage state and that they also provide information about joint cartilage wear so medical doctors can predict when it is necessary to perform surgery on a patient. In this research, we apply various kinds of sensors such as a system of infrared cameras and reflective markers, three-axis accelerometer, and force plate. The fluorescent marker and accelerometers are placed on the patient's hip, knee, and ankle, respectively. During a normal walk we are recording the space position of markers, acceleration, and ground reaction force by force plate. Measured data are included in the biomechanical model of the knee joint. Geometry for this model is defined from CT images. This model includes the impact of ground reaction forces, contact force between femur and tibia, patient body weight, ligaments, and muscle forces. The boundary conditions are created for the finite element method in order to noninvasively determine the cartilage stress distribution.

## 1. Introduction

Sports activities and daily routines such as standing, walking, running, jumping, and other recreational activities impose relatively large loads and movements on the human knee joint. These tasks could cause injuries and degenerations in the joint ligaments, menisci, cartilage, and bones. Thus, knowledge of in vivo joint motion and loading during functional activities is needed to improve our understanding of possible knee joint degeneration and restoration. Internal loadings of knee anatomical structures significantly depend on a lot of factors such as external loads, body weight, ligaments, strengths, and muscles forces.

In the paper [1] the knee implants were used to directly measure loads of participants during daily activities. In vivo knee measurements are very invasive and practically impossible for the case described in our study. There are many techniques for measuring an external variable which can be further used to create biomechanical and mathematical models.

Other studies [2] were based on the registration of fluoroscopic images and computer model of the knee. The most recent method utilized force plate data, CT or MRI skeletal structure data, and motion capture obtained from the infrared position sensor [3, 4]. In another research paper [5] the accelerometer was used to estimate the angle of lower extremities. This is a pretty cheap technique but requires additional processing of collected data and the error of estimated angle is up to six percent. The measure performance can be improved using a combination of accelerometer and gyroscope sensor such as goniometer [6–8].

In the study [9] stress on the knee cartilage during kneeling and standing using finite element models is compared. They used magnetic resonance (MR) images of the flexed knee to build a geometrical model. As a computational tool they used commercial software MIMICS. The results of those studies showed some differences in high-stress regions between kneeling and standing. The conclusion was that the peak von Mises stress and contact pressure on the cartilage were higher in kneeling. The study [10] used computed tomography (CT) images of knee structures during static loading to determine cartilage strains and meniscal movement in a human knee at different time periods of standing and to compare them with the subject-specific 3D finite element (FE) model. The results of these experiments showed that 80% of the maximum strain in cartilage developed immediately and after that cartilage continued to deform slowly. In the study [11] magnetic resonance (MR) images of the right knee of a 27-year-old male subject were used to determine the subsequent alteration in the fluid pressurization in the human knee using a three-dimensional computer model. The results of these studies indicated a redistribution of stresses within the tissue and a relocation of the loading between the tissue matrix and fluid pressure.

The purpose of this study was to estimate stress distribution in the knee cartilage. For that purpose the appropriate system of cameras and force plate platform were used. The deformation of cartilage was measured using marker position data and the 3D model of the lower leg segment. The models were established from computed tomography (CT) images. Simultaneously the deformation is assessed only matching single infrared camera images and CT image using software for image registration technique. In the experimental part the accelerometer sensor was used which potentially can provide more information about the gait. In the computer simulation the FEM analysis was applied with an adaptive change of mechanical parameters of tissue variation in order to match the measured force and deformation.

## 2. Materials and Methods

### 2.1. Mechanical Model of the Knee Joint

**Figure 1:**
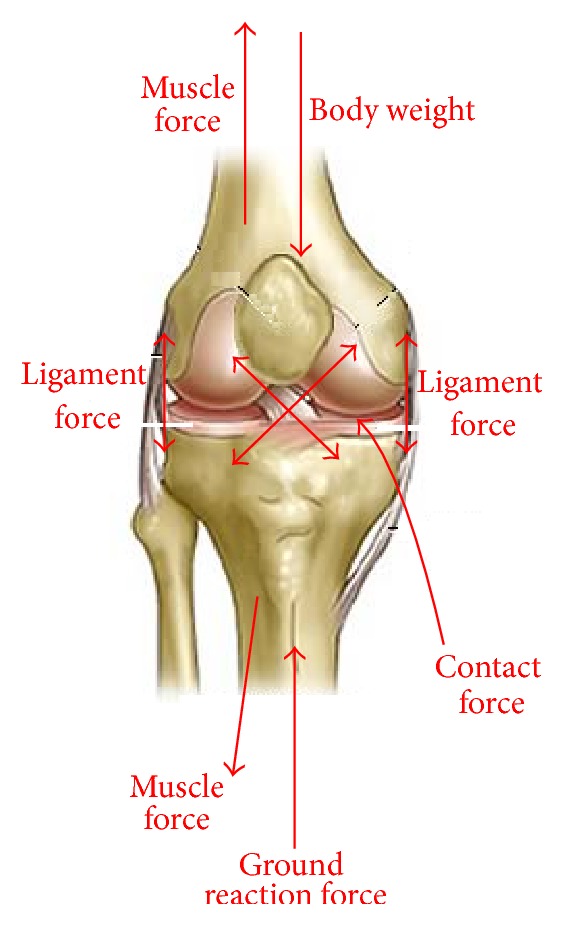
Forces on a knee joint.

Knee joint motion represents a complex combination of rotations and translations. The major parts involved in the knee kinematical behavior include femur, tibia and patella. Forces that act at a knee joint are given in Figure 1.

The dominant forces that act at a knee joint are body weight and ground reaction force which are opposite to each other. Muscle forces as well as contact forces between femur and tibia are also included in the model. Equilibrium equations of the knee joint are given below [5]:

(1) Fr+Fb+Fp1·N1+Fp2·N2+∑i=17Fi=0,Mr+Fr×Pr+Fb×Pb+Fp1·N1×P1+Fp2·N2×P2+∑i=17Fi×Vi=0,

where *F*_*r*_ is the ground reaction force, *F*_*b*_ is the body weight, *F*_*p*1_ and *F*_*p*2_ are the two contact point forces, *N*_1_ and *N*_2_ are the two contact points normal, *F*  (*i* = 1 ⋯ 7) are the ligament and capsule forces, *M*_*r*_ is the knee joint driver moment along local *z* axis and *P*_*r*_, *P*_*b*_, *P*_1_, *P*_2_ and *V*  (*i* = 1 ⋯ 7) are the position vectors where the corresponding forces are being applied [13]. We assumed that femur and tibia could be represented as rigid bodies and that their deformations were very small in contrast to relatively large deformations of cartilage and ligaments. Note that synovial fluid significantly reduces the friction between cartilage surfaces and menisci.

**Figure 2:**
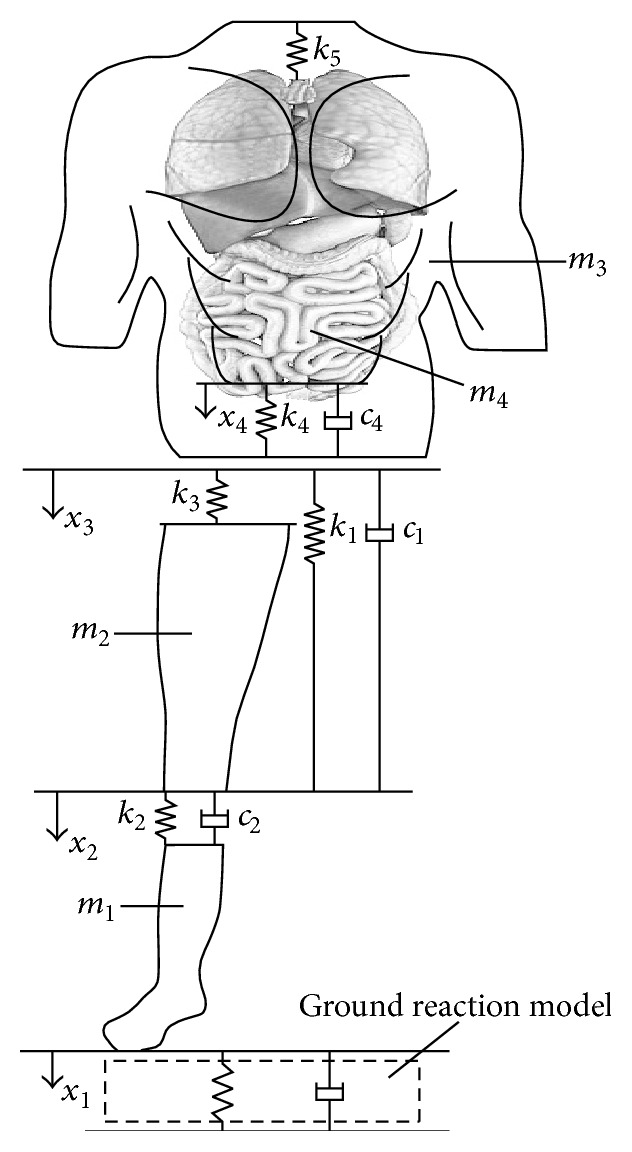
A simplified spring-damper-mass model used in the computer simulation. The components of the dynamics system presented are lower body rigid mass (*m*_1_) and wobbling mass (*m*_2_), upper body rigid mass (*m*_3_) and wobbling mass (*m*_4_), compressive spring (*k*_1_) and damper (*c*_1_) that connect the upper and lower rigid bodies, spring (*k*_3_) and spring}damper unit (*k*_2_, *c*_2_) connecting the lower wobbling mass to the upper and lower rigid bodies, and spring (*k*_5_) and spring}damper unit (*k*_4_, *c*_4_) connecting the upper wobbling mass to the upper rigid mass (adopted from [12]).

A simplified spring-damper-mass model which was used in this study is shown in Figure 2. It consists of four masses. The upper body was modeled using two masses, one representing its rigid mass, *m*_3_, and the other representing its wobbling masses, *m*_4_. The thigh, leg, and foot of the supporting leg were modeled using two masses, one representing its rigid mass, *m*_1_, and the other representing its wobbling masses, *m*_2_.

The total body mass was obtained from the participant. In the following system of equations (Equation (2)) a dynamics system is described [12] and was later used to calculate the resultant force and moment of the knee cartilage during the stance phase of the gait cycle:

(2) m1x¨1+k1x1−x3+k2x1−x2+c1x˙1−x˙3+c2x˙1−x˙2=m1g−Fg,m2x¨2−k2x1−x2+k3x2−x3+c2x˙1−x˙2=m2g,m3x¨3−k1x1−x3−k3x2−x3+k4+k5x3−x4−c1x˙1−x˙3+c4x˙3−x˙4=m3g.

In (2), *m*_1_ was the lower body rigid mass and *m*_2_ was the wobbling mass, *m*_3_ was the upper body rigid mass and *m*_4_ was the wobbling mass, *k*_1_ was the compressive spring and *c*_1_ was the damper that connected the upper and lower rigid bodies, *k*_3_ was the spring and *k*_2_-*c*_2_ was the spring-damper unit which connected the lower wobbling mass to the upper and lower rigid bodies, and *k*_5_ was the spring and *k*_4_-*c*_4_ was the spring damper unit which connected the upper wobbling mass to the upper rigid mass [12]. *F*_*g*_ was the vertical contact force which was defined as

(3) $$\begin{array}{c} F_{g}=\left\{\begin{array}{cc} A_{c}\cdot \left(ax_{1}^{b}+cx_{1}^{d}{\dot{x}}_{1}^{e}\right) & x_{1}>0,\\ 0 & x_{1}\leq 0. \end{array}\right. \end{array}$$

*A*_*c*_ is contact area and *a*, *b*, *c*, *d*, and *e* are the parameters of the ground reaction model which defined the deformation of the shoes during standing. Parameters for soft and hard shoes are shown in Table 1.

Cartilage was considered as a porous deformable body filled with fluid occupying the whole pore volume. The physical quantities for this analysis were the displacement of solid **u**, relative fluid velocity with respect to the solid (Darcy's velocity) **q**, fluid pressure **p**, and electrical potential *ϕ*. The governing equations for the coupled problem are described as follows. First, we considered the solid equilibrium equation:

(4) $$\begin{array}{c} \left(\;1-n\;\right)L^{T}\sigma_{s}+\left(\;1-n\;\right)\rho_{s}b+k^{-1}nq-\left(\;1-n\;\right)\rho_{s}\ddot{u}=0, \end{array}$$

where **σ**_*s*_ was the stress in the solid phase, *n* was porosity, **k** was the permeability matrix, *ρ*_*s*_ was the density of the solid, **b** was body force per unit mass, **q** was relative velocity of the fluid, and $\ddot{u}$ was acceleration of the solid material. The operator **L** ^*T*^ was

(5) $$\begin{array}{c} L^{T}=\left[\begin{array}{cccccc} \frac{\partial }{\partial x_{1}} & 0 & 0 & \frac{\partial }{\partial x_{2}} & 0 & \frac{\partial }{\partial x_{3}}\\ 0 & \frac{\partial }{\partial x_{2}} & 0 & \frac{\partial }{\partial x_{1}} & \frac{\partial }{\partial x_{3}} & 0\\ 0 & 0 & \frac{\partial }{\partial x_{3}} & 0 & \frac{\partial }{\partial x_{2}} & \frac{\partial }{\partial x_{1}} \end{array}\right]. \end{array}$$

The equilibrium equation of the fluid phase (no electrokinetic coupling) was

(6) $$\begin{array}{c} n\nabla p+n\rho_{f}b-k^{-1}nq-n\rho_{f}{\dot{v}}_{f}=0, \end{array}$$

where **p** was pore fluid pressure, *ρ*_*f*_ was fluid density, and was $\dot{v}$ fluid velocity. This equation is also known as the generalized Darcy's law. Both equilibrium equations were written per unit volume of the mixture. Combining (3) and (5) we obtain

(7) $$\begin{array}{c} L^{T}\sigma +\rho b-\rho \ddot{u}-\rho_{f}\dot{q}=0, \end{array}$$

where **σ** was the total stress which can be expressed in terms of **σ**_*s*_ and **p** as

(8) $$\begin{array}{c} \sigma =\left(\;1-n\;\right)\sigma_{s}-nmp, \end{array}$$

and *ρ* = (1 − *n*)*ρ*_*s*_ + *nρ*_*f*_ was the mixture density.

Here **m** was a constant vector defined as $m^{T}=\left\{\begin{array}{cccccc} 1 & 1 & 1 & 0 & 0 & 0 \end{array}\right\}$ to indicate that the pressure contributes to the normal stresses only. We also had to take into account the fact that the pressure has a positive sign in compression. Tensional stresses and strains were considered positive as well. In the following analysis we employed the effective stress, **σ**′, defined as

(9) $$\begin{array}{c} \sigma^{\prime }=\sigma +mp, \end{array}$$

which was relevant for the constitutive relations of the solid. Using the definition of relative velocity **q** as the volume of the fluid passing in a unit time through a unit area of the mixture (Darcy's velocity), we obtained

(10) $$\begin{array}{c} q=n\left(\;v_{f}-\dot{u}\;\right) \end{array}$$

and transformed (6) into

(11) $$\begin{array}{c} -\nabla p+\rho_{f}b-k^{-1}q-\rho_{f}\ddot{u}-\frac{\rho_{f}}{n}\dot{q}=0. \end{array}$$

The final continuity equation using the elastic constitutive law and fluid incompressibility was given in the form

(12) ∇Tq+mT−mTCE3Kse˙+1−nKs+nKf−mTCEm9Ks2p˙=0.

The resulting FE system of equations was solved incrementally [14] with a time step Δ*t*. We imposed the condition that the balance equations are satisfied at the end of each time step (*t* + Δ*t*). Hence, we derived the following system of equations:

(13) muu0000000mqu0000000u_¨t+Δtp_¨t+Δtq_¨t+Δtϕ_¨t+Δt+00cuq0cpucpp0000cqq00000u_˙t+Δtp_˙t+Δtq_˙t+Δtϕ_˙t+Δt+kuukup0000kpq00kqpkqqkqϕ0kϕp0kϕϕΔu_Δp_Δq_Δϕ_=Fut+ΔtFpt+ΔtFqt+ΔtFϕt+Δt,

where *F*_**u**_, *F*_**p**_, *F*_**q**_, and *F*_*ϕ*_ were forces in the balance equations for displacement, pressure, fluid velocity, and electrical potential, respectively, and **m**_**uu**_ and **m**_**qu**_ were mass terms in mass matrix [14].

### 2.2. Experimental Parts

**Figure 3:**
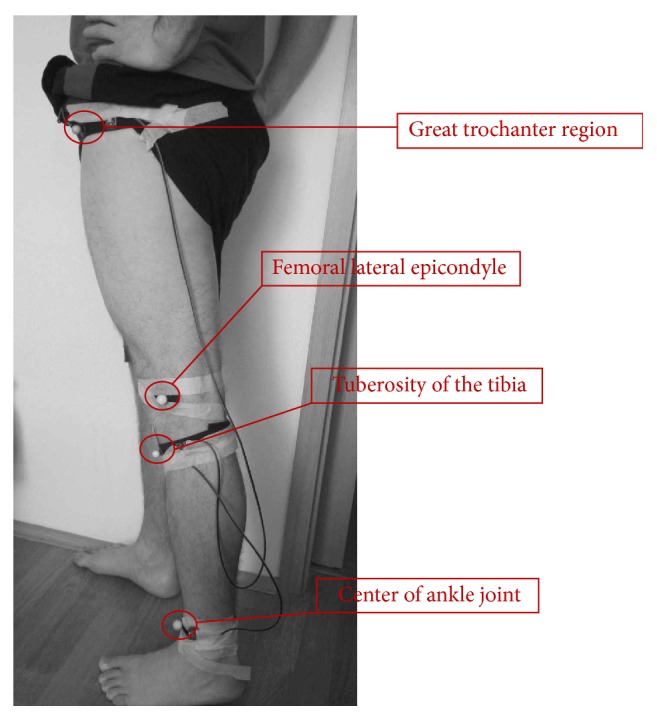
Reflective markers position on the examinee leg.

In this study we used a commercial motion capture system OptiTrack. This system consists of six infrared cameras and four retroreflective markers. The markers are 1.5 cm in diameter and are attached at the precise anatomical locations of the participant's leg for unilateral gain analysis. These locations were great trochanter region, femoral lateral epicondyle, tuberosity of the tibia, and the center of the anterior region of ankle joint (see Figure 3).

The computerized camera system with accompanying software captures the exact motion of retroreflective markers and thus records their trajectory while the volunteer performs walking over the force plate. The cameras were connected to a computer that collects gait kinematical data. The result of motion tracking is a series of 3D coordinates for each numbered marker. To better understand kinematics and kinetics of gait we used a three-axial accelerometer.

We used Sun SPOT accelerometers (Sun Small Programmable Object Technology) to wirelessly detect the middle of the gait stance phase, that is, the moment when the ground reaction force reaches its maximum value. The Sun SPOT has a sensor board which consists of 2G/6G 3-axis accelerometer, temperature sensor, and light sensor. In the experimental part the Sun SPOT LIS3L02AQ accelerometer is used to measure the orientation or motion in three dimensions, *X*, *Y*, and *Z* with the sample rate of 100 Hz. Each of these components represents the sum of static accelerations defined by angle of inclination to the corresponding axis and dynamics component stemming from the movement during the walk. The ground reaction force was sampled from the multiaxis AMTI force plate at the rate of 100 Hz. Before the experiment, calibration was conducted to work out the space coordinate system for the camera system field of view. The calibration was performed fully in accordance with the proposed manufacturer's procedure.

**Figure 4:**
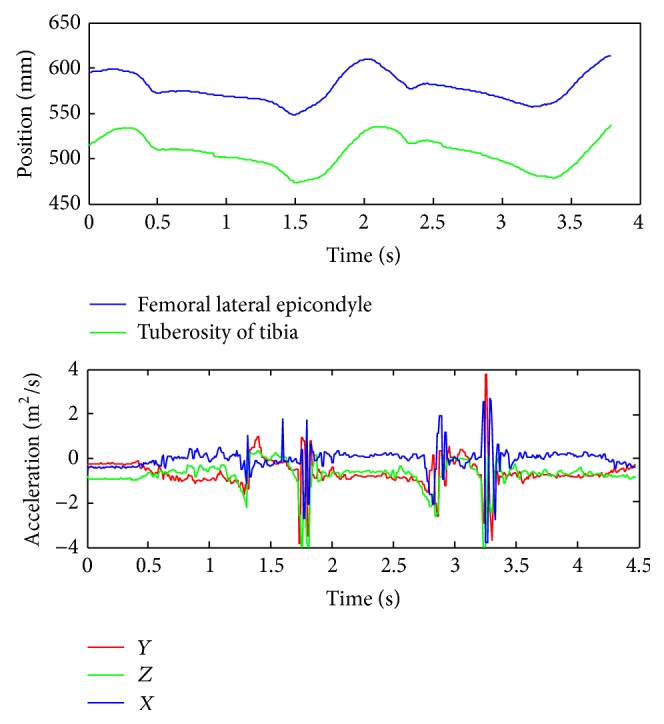
Position of reflective marker during walking and corresponding three-axial acceleration.

The volunteer performs walking along 2.5 meter distance path away with his own ordinary velocity and attached infrared marker and accelerometers sensor on the left leg. Results for marker coordinates and corresponding accelerations, measured by the three-axial accelerometer, are presented in Figure 4.

According to [15–17] measured values for marker position are influenced by noise due to the wobbling of the participant's skin. The value of this uncertainty is in range ±2 mm.

**Figure 5:**
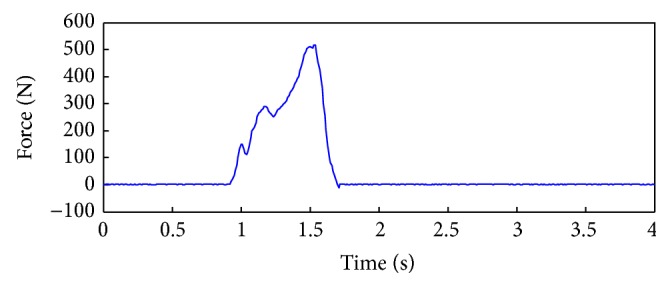
Ground reaction forces during standing on the force plate.

The force plate is positioned in the first half of the walking path. During the experiment, the participants were asked to walk along so the force plate records the value of the ground reaction force (Figure 5).

The force value is zero in the beginning of the walk and when the participant stands on the force plate, starting with the heel, the force gradually increases and reaches the maximum and then drops to zero again when the foot is detached by the force plate.

## 3. Results

One of the main tasks for preparing data for FEM simulation is matching the infrared reflective marker position obtained by infrared camera and landmark position on the CT images when cartilage is unreformed. This procedure is known as image registration and ANTs (Free software for image registration) is used [18].

**Figure 6:**
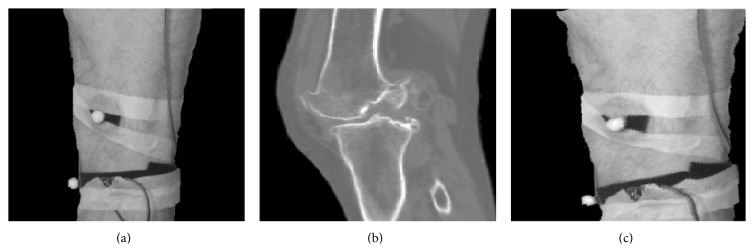
Registration infrared image and CT image. (a) Infrared camera image; (b) CT image; (c) registered image.

Main goal of the registration process is obtained from the transformation parameter that maps the marker position in deformed and undeformed knee cartilage. In general this process is finding the optimal transformation that maps every pixel of image with the presence of deformation and another static image when cartilage is undeformed. As similarity measure of image the mutual information is used. The standard algorithm in registration process is elastic deformation procedure. The idea is to model the contours on one of the matching images as elastic object which deformed under the influence of some external forces [19]. In each step of the deformation the images are compared and value of external forces is now proportional to the difference between these cases on the basis of mutual information value. The process is repeated until the difference between the images is greater than some error of convergence. The result of registration between CT and infrared images is shown in Figure 6. The measured difference of the marker positions between deformed and undeformed cartilage is 1.78 ± 0.6 mm. Images showing markers matching these cases are presented in Figures 6(a) and 6(c), respectively. The measured uncertainty of 0.06 mm corresponds to the dimension of a single pixel. The error in estimation of the deformation is significantly greater due to the influence of the distortion of the camera lens, skin movements, and insufficiently accurate registration result.

Simultaneously, we create a 3D model using CT slices. The CT slices are segmented using a threshold value and compared with gray value of the image pixel. The segmented images are submitted by the edge detection operator for boundary extraction. The merging of adjacent boundary is done in each slice and final 3D model is created (Figure 2). This model includes femur, tibia, cartilage, surrounding tissue, and skin.

**Figure 7:**
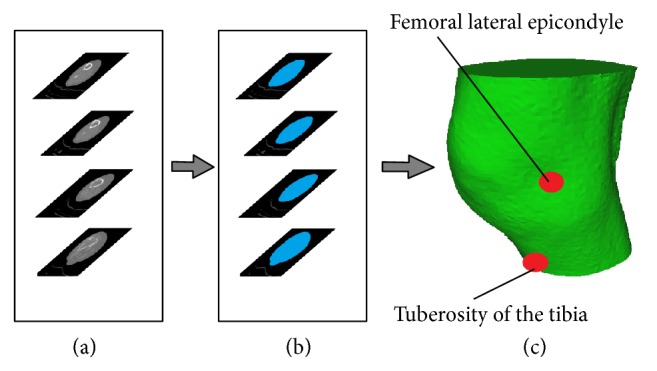
3D model of knee. (a) Raw CT slices; (b) segmented CT images; (c) 3D model with marker position.

The anatomical point (tuberosity of tibia and femoral lateral epicondyle) can be easily detected in the model and initial position of the marker can be obtained. Using the measured position of infrared marker (Figure 4) and anatomical position of the marked point on the created 3D model (Figure 7(c)) we can obtain a vertical deformation of cartilage as difference of these values. The observed measured value coincides with the point when the ground reaction force is in the maximum. According to Figure 5 this moment is 1.49 seconds after starting the recording of gait and the deformation is 2.30 ± 0.01 mm. The measured uncertainty of 0.01 mm emerges as a consequence of the limited resolution of the motion capture camera system and resolution of the CT scanner. This methodology for obtained deformation is more precise than registration method but it requires more processor time and memory.

Using the same procedure for image segmentation, a full model of knee joint is created. The model consists of femur, tibia, and cartilage.

**Figure 8:**
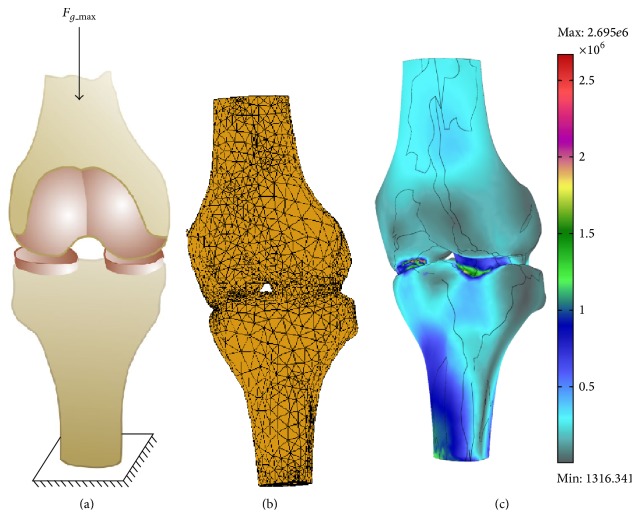
FEM analysis model. (a) Model filled with the tetrahedral mesh element; (b) knee von Misses stress distribution in [Pa].

We used measured values for the displacement and ground reaction force in order to calculate corresponding matrix elements in the relation (13). Upon model creation we applied boundary conditions: (a) we clamped the distal end of tibia and (b) axially loaded the femur with the maximally measured ground reaction force *F*_*g*_max⁡_ = 511 N (Figure 8(a)).

For modeling the cartilage and meniscus we implemented a finite element formulation where the nodal variables are displacements of solid, **u**; fluid pressure, **p**; Darcy's velocity, **q**; and electrical potential, *ϕ* with estimated matrix elements. A standard procedure of integration over the element volume was performed and the Gauss's theorem was employed. An implicit time integration scheme was implemented.

The tetrahedral mesh model had 20537 elements and 4693 nodes (Figure 8(b)). We used PAK solver [20] for the FEM analysis. Total execution time of the analysis was around 30 minutes on the core I7 processor with 12 GB of RAM memory.

The initial mechanical characteristic, Young's modulus, and Poisson's ratio, for the femur and tibia, were amounted to *E* = 20 GPa and *ν* = 0.3, for the isotropic cartilage *E* = 10 MPa and *ν* = 0.45, and for the transversely isotropic menisci *E* = 20 MPa and *ν* = 0.3. All these values were taken from the literature [21].

These values were adaptively changed for the purpose of correspondence between the measured deformation and ground reaction force.

The final value of the Young's modulus of the cartilage is 5.62 MPa with error of estimation of 0.01 MPa. The Young's modulus for the femur and tibia is adapted to the 18 GPa while the Poison's ratio is 0.45.

Resultant stress distribution of the FEM analysis for the elements of the knee joint is given in Figure 8(c).

**Figure 9:**
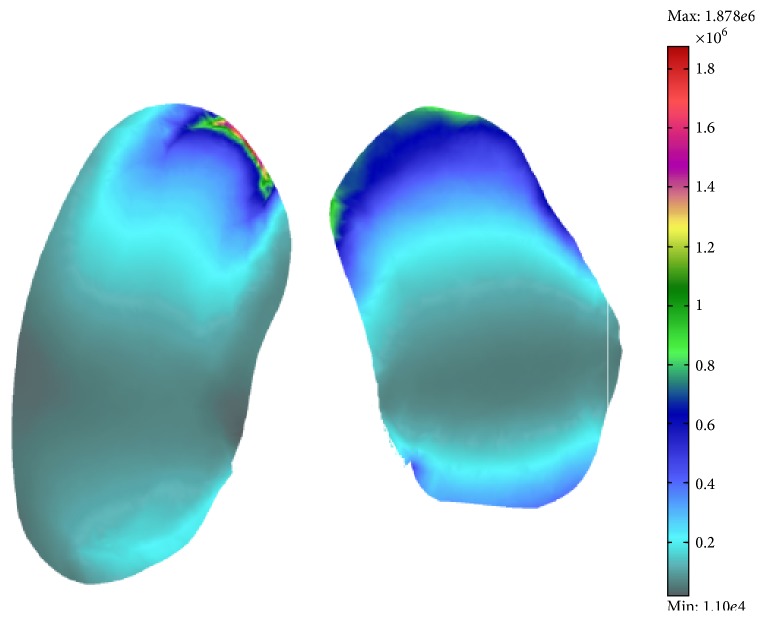
Knee cartilage von Misses stress distribution [Pa].

The von Mises stress on the cartilage is presented in Figure 9. As it can be seen the maximal value of stress has MPa magnitude of order and is located on the boundary of the cartilage. This is in compliance with the fact that is cartilage is the weakest part of the knee joint with a tendency to injury and fraying.

The procedure described in this study can offer very useful information for physicians in order to better understand injury formation and improve the process of rehabilitation and, in some perspective, as a support in clinical decision making.

## 4. Conclusion

The main goal of this study was to introduce a new approach towards a noninvasive effective stress calculation for a specific participant. Input data were provided from the experimental measurements during the participant's walking test whereupon finite element analysis was performed giving the distribution of the effective stress in the major anatomical elements of the knee joint: femur, tibia, and cartilage. This approach demonstrates a great possibility for preoperative and postoperative surgical planning and treatment of the knee injuries for specific patients.

This study contains some limitations. We neglected the skin movement artifact during the experiment and the influence of ligament presence. However, the current model allows us to investigate the effect of different biomechanical factors and loads on the stress distribution at the knee joint. In this study we used data obtained from infrared cameras and force plate sensors. Besides, in our future work we will try to replace a relatively expensive system of infrared cameras with a much cost effective system of accelerometers so that we will be able to calculate positional data of anatomical points of the lower extremities solely by using their accelerations. The very promising are techniques of the image registration that can be used for assessment of gait parameter using a cheaper mobile cell camera. This will be a consideration of the future work.

## Figures and Tables

**Table 1:** Parameter for ground reaction model.

	*a*	*b*	*c*	*d*	*e*
Soft shoe	10^6^	1.56	2 × 10^4^	0.73	1.0
Hard shoe	10^6^	1.38	2 × 10^4^	0.75	1.0
